# Hydrogenation and Entanglement Effects on Tensile Properties and Energy Absorption of Entwined Coiled Carbon Nanotubes: A Molecular Dynamics Study

**DOI:** 10.3390/ma19050943

**Published:** 2026-02-28

**Authors:** Fenghua Nie, Xing Su, Hang Lin

**Affiliations:** School of Resources and Safety Engineering, Central South University, Changsha 410083, China; fenghuanie@csu.edu.cn (F.N.); xingsu23@csu.edu.cn (X.S.)

**Keywords:** entwined coiled carbon nanotubes, hydrogenation, tensile properties, molecular dynamics simulations, energy absorption

## Abstract

Coiled carbon nanotubes and their entwined derivatives have attracted attention for their excellent mechanical properties and potential applications. Hydrogenation is an effective way to modify the mechanical response of CCNTs, but its effect on the tensile performance and energy absorption of ECCNTs remains unclear. In this study, molecular dynamics simulations are used to systematically investigate the regulatory effects of hydrogenation and entanglement on the tensile properties and energy absorption capacity of CCNTs and ECCNTs. Different hydrogenation degrees and structural configurations are designed for comparative analysis. Results show that hydrogenation exerts a dual regulatory effect on single CCNTs: moderate hydrogenation enhances tensile strength and total energy absorption, while excessive hydrogenation leads to brittleness, reduced ductility, and impaired energy dissipation. Entanglement significantly improves the tensile load-bearing capacity and energy absorption of CCNTs, with ECCNTs showing 1.5–2.0 times higher spring constants than single CCNTs. Among the ECCNT models, ECCNT2 achieves an optimal balance between tensile stiffness, ductility, and energy absorption efficiency. This study clarifies the mechanistic roles of hydrogenation and entanglement, providing valuable guidance for the rational design of high-performance CCNT-based structural and functional nanomaterials.

## 1. Introduction

Coiled carbon nanotubes (CCNTs) represent a distinctive class of nanostructures known for their helical or spring-like shape, providing exceptional elasticity, resilience, and mechanical tunability compared to traditional straight carbon nanotubes (CNTs) [1]. Their unique coiled structure allows for significant reversible deformations, making them highly promising for applications in flexible electronics, energy absorption, and nanoscale actuators [2,3]. When multiple CCNTs intertwine or entangle, entwined coiled carbon nanotubes (ECCNTs) can be formed. This configuration introduces additional mechanical complexity due to the interactions and friction among the nanosprings [4]. These hierarchical configurations exhibit enhanced load-bearing capacity and energy dissipation properties, thereby opening up new possibilities for the design of high-performance, deformable nanomaterials.

Hydrogenation has emerged as a powerful strategy for modifying the mechanical response of CCNTs by altering their local bonding configurations and surface chemistry [5]. At low levels of hydrogenation, the adsorption of hydrogen atoms transforms portions of the rigid, planar sp^2^ carbon network into more flexible sp^3^ bonds [6]. This rehybridization induces local wall distortions and weakens the load-bearing structure of the coiled nanotubes, resulting in lower stiffness properties such as reduced Young’s modulus and spring constant [7]. The decline in stiffness becomes especially significant when the hydrogen atoms are distributed randomly, as this irregular arrangement amplifies curvature irregularities and generates localized structural defects along the helical walls, causing a steep early reduction in axial rigidity even with hydrogen coverage below approximately 10% [8]. Furthermore, it is revealed that when functionalization exceeds a certain threshold—generally around 30% surface coverage—hydrogenation can enhance the stretchability and deformability of CCNTs. This modification allows for the larger recoverable tensile strains compared to pristine springs, while also maintaining relative insensitivity to temperature changes in terms of elastic constants [9]. Recent research on HCCNTs suggests that hydrogenation not only modifies the intrinsic tensile behaviors but also improves the interfacial interactions and energy dissipation mechanisms when HCCNTs are incorporated into the matrices [10]. However, most existing studies have concentrated on isolated coils or simple nanocomposite pullout scenarios, rather than on the complex, architected entwined networks [11,12]. The effect of hydrogenation on the tensile performance of structurally intricate systems, such as entwined CCNT assemblies—where the mechanical response is influenced by the coil deformation, intercoil contact, and frictional sliding—remains largely unexamined. Addressing this critical gap, this study systematically explores how hydrogenation and entanglement jointly impact the tensile behaviors and energy absorption capacities of both individual CCNTs and ECCNT structures, offering new insights into the interplay between hydrogenation and entanglement in determining their mechanical performance.

Molecular dynamics (MD) simulations offer a robust atomistic framework for investigating the mechanical properties of nanomaterials, since they can effectively capture the bond breaking, defect evolution, and interfacial contact and sliding during the structural deformations [13,14,15,16,17]. The experimental and continuum-based investigations have provided valuable insights into the deformation, fracture, and interfacial mechanical responses of engineering materials [18,19,20]. Unlike the continuum models, MD inherently accounts for the effects of local curvature, chirality, and functionalization on the mechanical responses, which is crucial for accurately representing the complex, hierarchical geometry of ECCNTs. In recent decades, MD methods have been extensively and successfully utilized to explore the elastic, plastic, and failure characteristics of both straight and coiled carbon nanotubes, providing quantitative insights into the size effects, loading-rate dependence, and temperature sensitivity that are challenging to obtain experimentally at the nanoscale [21,22,23]. Building on these established advantages, MD simulations are employed as the primary tool to analyze the tensile response of both individual CCNTs and their entwined configurations. This approach facilitates a comprehensive mechanistic understanding of how hydrogenation, coil architecture, and intercoil interactions collectively influence the macroscopic load-bearing and energy-absorbing capabilities of ECCNT systems.

The objective of this study is to elucidate how hydrogenation modulates the tensile performance of both individual CCNTs and their entwined assemblies, with a particular focus on the interplay between chemical functionalization and hierarchical coil architecture. To address this objective, MD simulations are performed to systematically tailor hydrogen coverage and structural configurations, enabling direct comparative analysis of CCNTs, HCCNTs, and ECCNTs under uniaxial tensile loading. Results reveal that hydrogenation exerts a dual regulatory effect on the tensile properties of CCNTs: moderate hydrogenation enhances tensile strength and total energy absorption by improving structural distortion tolerance of the sp^2^–sp^3^ hybrid network, while excessive hydrogenation induces severe structural brittleness, leading to reduced ductility and compromised energy dissipation capacity. Additionally, the entanglement of CCNTs drastically augments their tensile load-bearing capacity and energy absorption performance, which stems from the synergistic inter-tube interactions and additional energy dissipation paths introduced by the hierarchical entwined architecture. These findings provide mechanistic insights for the rational design of hydrogenated ECCNT-based structural and functional materials with optimized tensile and energy absorption properties, and further highlight the great potential of MD simulations as a predictive tool for the design and optimization of architected nanotube systems.

## 2. Model and Methodology

### 2.1. Atomistic Model

**Figure 1:**
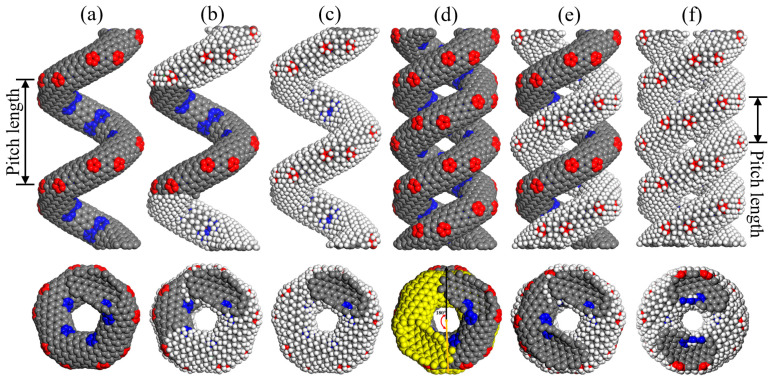
Molecular structures of (**a**) CCNT, (**b**) HCCNT1, (**c**) HCCNT2, (**d**) ECCNT1, (**e**) ECCNT2, (**f**) ECCNT3 in the front and vertical view. (The carbon and hydrogen atoms are shown in grey and white colors, while the pentagon and heptagon carbons are presented in red and blue colors. The yellow segment in ECCNT1 represents the 180° entanglement conformation of CCNT1).

The molecular structures of different CCNTs and ECCNTs are depicted in Figure 1. To create the desired geometric shapes of CCNTs, heptagon and pentagon defects are introduced at different hexagonal positions within the CNTs, as denoted by the red and blue colors in Figure 1a. The detailed construction methods of the CCNT molecular structures have been thoroughly described in our previous study [24], including the specific positions of heptagon and pentagon defects and the formation mechanism of the helical structure. Figure 1b presents the molecular structure of HCCNT1, which is created by hydrogenating half of the carbon atoms in the upper and lower CCNT structures. In contrast, Figure 1c shows the full hydrogenation model of HCCNT2, where the hydrogenation coverage rate is 100%. Moreover, 50% hydrogenation is chosen to represent moderate functionalization that maintains a balanced sp^2^–sp^3^ hybrid network, while 100% hydrogenation serves as the saturated limit to reveal the brittle response caused by excessive sp^3^ bonding. Leveraging these homogeneous hydrogenation distributions, we effectively eliminate the statistical variability associated with random doping, thereby enabling a clear and unambiguous comparison of how increasing sp^3^ hybridization influences the stiffness, ductility, and failure mode of the nanostructures. From an experimental perspective, the sectional hydrogenation coverage selected in our models is representative of site-selective or patterned hydrogenation strategies—approaches that are becoming increasingly feasible through advanced techniques such as plasma treatment and atomic layer deposition [25,26]. These state-of-the-art methods facilitate the precise functionalization of specific surfaces or regions within nanostructures, rendering our theoretical models highly relevant to emerging experimental endeavors aimed at tailoring mechanical properties via spatially controlled chemical modification [27]. Additionally, three ECCNT models are depicted in Figure 1d–f. As illustrated in Figure 1d, the ECCNT1 model is created by entwining two CCNT structures, with one of the CCNTs twisted 180 degrees at the location of the other. A 180° twist ensures symmetric and uniform contact between the two coiled nanotubes, avoiding asymmetric structural bias that might complicate the interpretation of mechanical responses. The 180° configuration has been widely adopted in previous studies of the intertwined CCNT systems, facilitating reasonable comparison with existing literature [4]. Building upon ECCNT1, ECCNT2 is formed by hydrogenating one of the CCNTs, while ECCNT3 employs hydrogenation on both CCNTs. All the nanosprings used in this study are single-walled nanotubes, initially positioned at the center of a simulation box measuring 60 Å × 60 Å × 60 Å. The pitch length is defined as the axial distance corresponding to one complete helical turn of a coiled carbon nanotube, as shown in Figure 1. All the models are constructed in Materials Studio 2020.

The geometrical parameters of the different nanosprings or entwined nanosprings are presented in Table 1. It can be checked that the tube radii of all nanosprings range from 4 Å to 5 Å. The pitch length of ECCNTs is reduced to half that of CCNTs and HCCNTs, mainly due to the intertwining effect, which decreases the distance between each pitch.

### 2.2. Forcefield

The Adaptive Intermolecular Reactive Empirical Bond Order (AIREBO) potential is adopted in this study, which has been extensively employed as the interatomic potential for carbon-based materials [28,29]. In this context, the interatomic potential energy within the AIREBO framework is computed using three primary components:

(1) $$E=\frac{1}{2}\sum_{i}\sum_{j\neq i}\left[E_{ij}^{REBO}+E_{ij}^{LJ}+\sum_{k\neq i,j}\sum_{l\neq i,j,k}E_{kijl}^{TORSION}\right]$$

where the first component, $E_{ij}^{REBO}$, representing the hydrocarbon Reactive Empirical Bond Order (REBO) potential [30], is defined as:

(2) $$E_{ij}^{REBO}=V_{ij}^{R}\left(r_{ij}\right)+b_{ij}V_{ij}^{A}\left(r_{ij}\right)$$

where the second component, $E_{ij}^{LJ}$, accounts for non-bonded interactions, modeled using the Lennard-Jones potential for distances ranging from 2 Å to the cutoff distance:

(3) $$E_{ij}^{LJ}=4\varepsilon \left[{\left(\frac{\sigma }{r}\right)}^{12}-{\left(\frac{\sigma }{r}\right)}^{6}\right]$$

where the *r* denotes the distance between interacting atoms. In addition, the standard AIREBO potential incorporates the term, $E_{kijl}^{TORSION}$, which is a 4-body potential designed to represent dihedral angle preferences in hydrocarbon structures. The cutoff distance in the switching function of the short-range REBO potential is increased to 2.0 Å to mitigate the unphysical strain hardening effects observed at extreme elongation levels [31]. Meanwhile, the cutoff parameter for the two-body 12–6 Lennard-Jones potential is set to 10.2 Å. These cutoff parameters have been widely adopted and validated in existing research for carbon-based nanostructures, including CCNTs, HCCNTs, and their entwined derivatives [4,8,32,33]. Three independent simulations with different random seed numbers are performed to ensure statistical consistency.

### 2.3. Simulation Method

Before the tensile test, the geometry optimization of CCNTs and ECCNTs structures is conducted using a conjugate gradient algorithm under an energy and force tolerance of 1.0 × 10^−5^ eV and 1.0 × 10^−6^ eV/Å, respectively. Once the optimization is complete, the systems are equilibrated for 500 ps within an NPT ensemble, maintaining a constant pressure of 1 atm and a temperature of 1 K to ensure the proper relaxation. The pressure and temperature control is achieved through a Nosé–Hoover barostat and a Nosé–Hoover thermostat, with damping times of *τ*_P_ = 1000 timesteps and *τ*_T_ = 100 timesteps, respectively. MD simulations are performed at 1 K mainly to minimize the thermal fluctuations and exclude the interference of thermal vibrations, so that we can clearly reveal the intrinsic mechanical deformation mechanisms, bond evolution, and structural failure modes of CCNTs and ECCNTs under tensile loading. This low-temperature setup of 1 K has been widely adopted in atomistic simulations of CNT-based materials to highlight the structure–property relationship without thermal noise [2,32,34]. During the relaxation phase, the periodic boundary conditions (PBC) are applied to the nanosprings along their axial direction to accommodate the length changes during the relaxation process. Following the relaxation process, the nanosprings are subjected to elongation at a constant strain rate of 10^8^/s within an NVT ensemble, which maintains the constant volume and temperature. The strain rate of 10^8^ s^−1^ employed in this work is widely adopted in molecular dynamics simulations of coiled carbon nanotubes and nanosprings [2,4,32,35]. Although this rate is several orders of magnitude higher than experimental loading rates, such a high strain rate is computationally necessary to achieve sufficient deformation within feasible simulation timescales. Temperature control is again controlled by a Nosé–Hoover thermostat. Tensile deformation is achieved by uniformly rescaling the coordinates of all atoms along the axial direction every 1000 timesteps until the complete rupture occurs. A time step of 1 fs is utilized, employing the velocity Verlet scheme to integrate the equations of atomic motion throughout all MD simulations. The strain of nanosprings is calculated as follows:

(4) $$\varepsilon =\frac{\left(l_{z}-l_{0}\right)}{l_{0}}$$

The pulling stress and forces are determined using the virial stress tensor components for each atom during the MD simulations. The effective cross-sectional areas for both CCNTs and ECCNTs are defined as follows:

(5) $$s=2\pi \bar{r}(d+t)$$

where *d* and *t* denote the diameter and wall thickness (3.35 Å) of the coil tubes, respectively. The effective cross-sectional area physically represents the intrinsic load-bearing cross-section of the nanotube wall material—a standard definition widely adopted in high-fidelity MD simulations of CCNT systems [2,4,32,35].

The effective radius of CCNTs and ECCNTs can be calculated as follows:

(6) $$\bar{r}=\frac{1}{N}\sqrt{\sum_{i=1}^{N}\left({\left(x_{i}-x_{center}\right)}^{2}+{\left(y_{i}-y_{center}\right)}^{2}\right)}$$

where *x_center_* and *y_center_* represent the center coordinates of CCNTs and ECCNTs in the *x*-*y* plane. *x_i_* and *y_i_* denote the coordinates of the position of the *i*-th atom in the same plane, and *N* refers to the atom number.

## 3. Results and Discussion

### 3.1. Single CCNT/HCCNT

**Figure 2:**
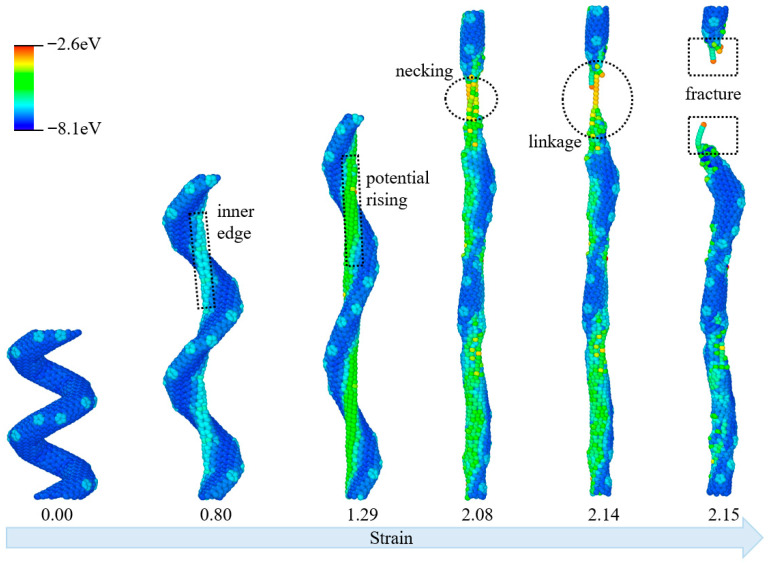
Structural evolutions of CCNT during the tensile process. (The atoms are colored based on their potential energy.).

The molecular deformations of CCNT are illustrated in Figure 2, with colors representing per-atom potential energy, ranging from red (indicating higher energy) to blue (indicating lower energy) throughout the tensile process. The CCNT exhibits a distinct helical morphology, with uniformly low potential energy predominantly represented in blue across all atoms. As tensile force is applied and the strain reaches 0.80, the potential energy first rises, shifting to green and yellow at the inner edges of the helical structure, while the outer edges retain their original low-energy state in blue. This trend corresponds to the initiation of structural distortion in the geometrically constrained inner regions. As the strain increases to 1.29, the helical curvature of the CCNT gradually diminishes, consistent with axial stretching, and the region of elevated potential energy expands along the tube. By a strain of 2.08, the morphology of the CCNT approaches that of a straight carbon nanotube. Critically, necking, characterized by localized narrowing and marked by a concentrated region of high potential energy in yellow and red, emerges in the upper segment, signaling the onset of partial structural failure as localized atomic bonds undergo excessive deformation. With subsequent loading at a strain of 2.14, the linkage between the necked fragments weakens; the high-energy necking region shrinks, leaving only a narrow atomic connection between the two segments, while the rest of the structure reverts to lower potential energy in blue and green. Finally, at a strain of 2.15, the linkage completely fails, resulting in the two segments detaching and a clear fracture forming, leading to the full rupture of the CCNT.

**Figure 3:**
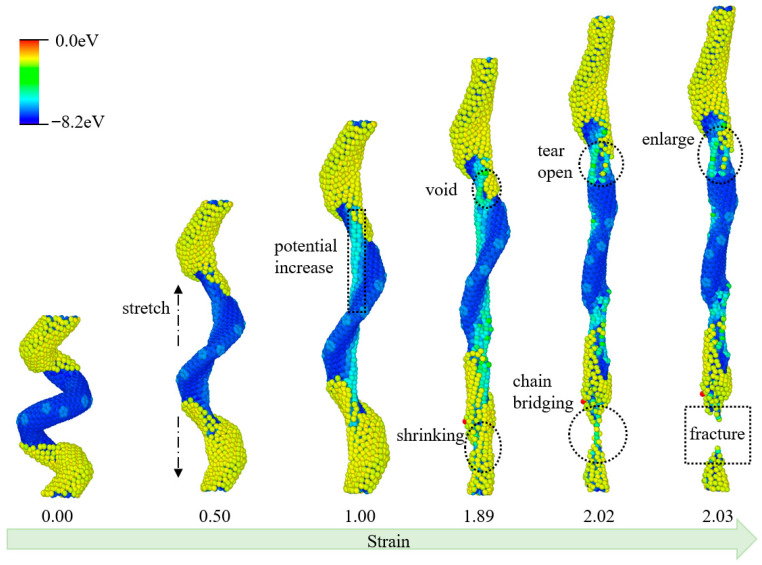
Structural evolutions of HCCNT1 during the tensile process. (The atoms are colored according to their potential energy.).

The molecular deformation of HCCNT1 during the tensile process is illustrated in Figure 3. A clear interface is present on the HCCNT1 surface between the hydrogenated CCNT and the pristine CCNT; the former exhibits a higher initial potential energy, while the latter has a lower initial potential energy. This interface defines distinct structural regions from the start, at a strain of 0.00, where the material maintains a compact helical morphology. As tensile force is applied, the potential energy increases from the inner edge of HCCNT1, evident by the expansion of yellow at a strain of 0.50. Notably, no apparent fracture occurs before a strain of 1.0; instead, the structure elongates with the gradual potential elevation concentrated at the inner helical edges. In contrast to the CCNT, some voids appear at the interfaces of the hydrogenation at a strain of 1.89, accompanied by a shrinking phenomenon in the hydrogenation region. Here, the hydrogenated segment contracts, while the unhydrogenated region retains relative structural integrity. As the tensile strain increases to 2.02, the void at the hydrogen interface further tears apart, while a chain bridging forms in the hydrogenated section. This temporary atomic linkage slows immediate fracture by connecting the separated hydrogenated sub-segments. With the continued increase in tensile strain to 2.03, fractures occur in the hydrogenation section, and the voids continue to enlarge throughout the tensile process. The hydrogenated region fragments, and the unhydrogenated segment elongates as the structure nears complete failure.

**Figure 4:**
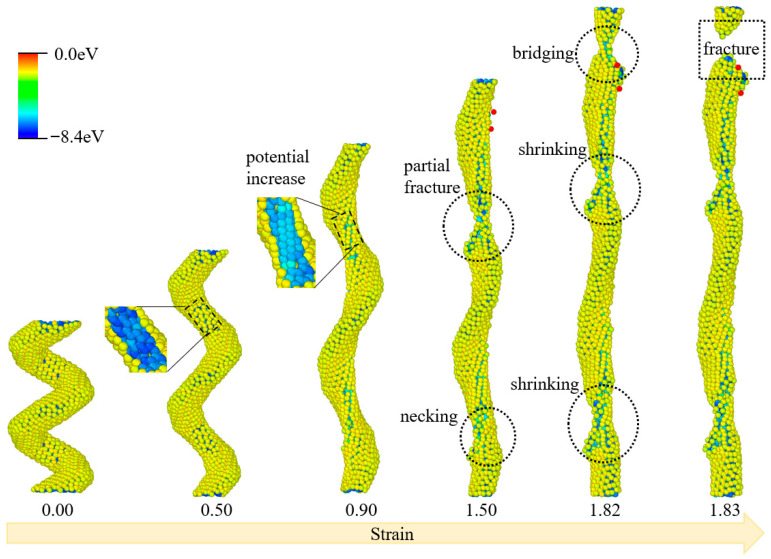
Structural evolutions of HCCNT2 during the tensile process. (The atoms are colored according to their potential energy.).

The structural evolution of HCCNT2, colored according to the per-atom potential energy during the tensile process, is depicted in Figure 4. Initially, at a strain of 0.00, HCCNT2 exhibits a compact helical morphology with uniform potential energy, predominantly yellow, consistent with its hydrogenated structure. A similar phenomenon is observed as the potential of the inner edge atoms increases when the strain rises from 0.5 to 0.9. At a strain of 0.50, localized potential elevation initiates at the inner helical edges, and this high-energy region expands along the inner structure by a strain of 0.90, reflecting early structural distortion at geometrically constrained sites. At a strain of 1.5, two distinct necking and fracture sections are visible in the middle and lower parts of HCCNT2. The middle region shows necking, characterized by localized narrowing with concentrated high potential energy, while the lower section displays partial fracture, indicated by a small, detached fragment. As the tensile strain continues to increase to 1.82, a significant fracture appears in the upper section of HCCNT2, accompanied by bridging, which consists of temporary atomic connections between fragmented segments. The middle section remains relatively unchanged, while the lower section gradually shrinks, with two regions where the hydrogenated structure contracts further. Finally, the upper part undergoes complete fracture when the strain reaches 1.83, as the bridging connections fail and the upper segment detaches fully.

**Figure 5:**
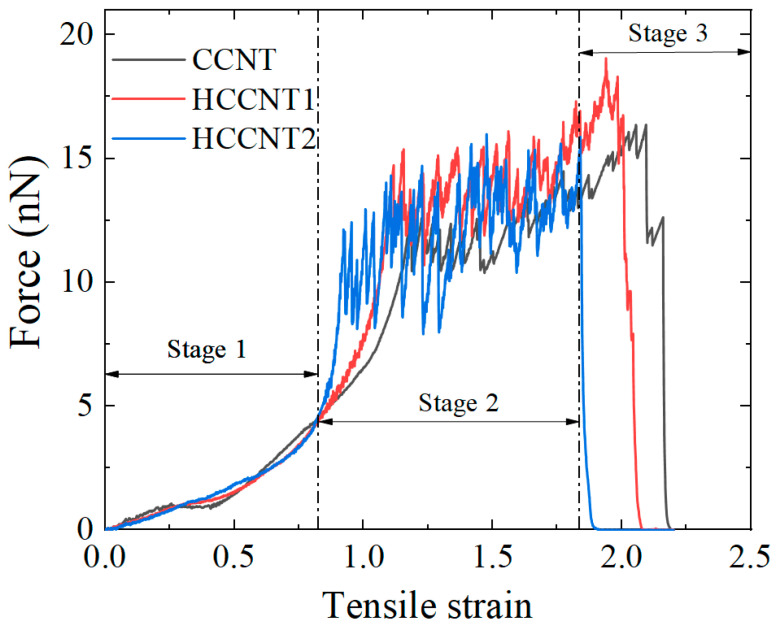
Force–strain curves of CCNT, HCCNT1 and HCCNT2 during the tensile process. The tensile process can be divided into three distinct stages: the increasing stage, the oscillation stage, and the decreasing stage.

The force–strain curves of CCNT, HCCNT1, and HCCNT2 are illustrated in Figure 5. Three stages can be observed from the strain-force curves as the increasing stage, the oscillation stage, and the decreasing stage, respectively. Up to a tensile strain of 0.8, the increasing trend of all three curves is almost the same and no significant differences are observed among the tensile curves during the increasing stage. It indicates that hydrogenation has a limited effect on tensile performance during the small-amplitude tensile tests. There is a strong oscillation period between 0.8 and 1.9, which has also been found in previous studies [32]. These oscillations arise from the incremental buckling and breaking of joints, which can be demonstrated through the evolution of molecular configurations under the tensile test from Figure 2, Figure 3 and Figure 4. However, the ultimate tensile strain decreases from 2.2 to 1.8 as the hydrogenation rate increases, suggesting that hydrogenation negatively impacts the structural ductility. Notably, the peak tensile force of HCCNT1 reaches 19 nN, which is higher than the 16.3 nN of CCNT and the 15.8 nN of HCCNT2. This suggests that the low-to-moderate hydrogenation enhances tensile strength and energy absorption efficiency, whereas excessive hydrogenation induces structural brittleness and degraded mechanical performance. The apparent strengthening of HCCNT1 does not arise from stronger individual bonds, but from the altered structural mechanics. The sp^2^–sp^3^ hybrid network enhances deformation tolerance and stress redistribution, yielding a higher peak force despite the local weakening of carbon bonds. This understanding of the structure–property relationship is valuable for the rational design of hydrogenated CNT-based materials with improved mechanical performance.

### 3.2. Entwined CCNTs/HCCNTs

**Figure 6:**
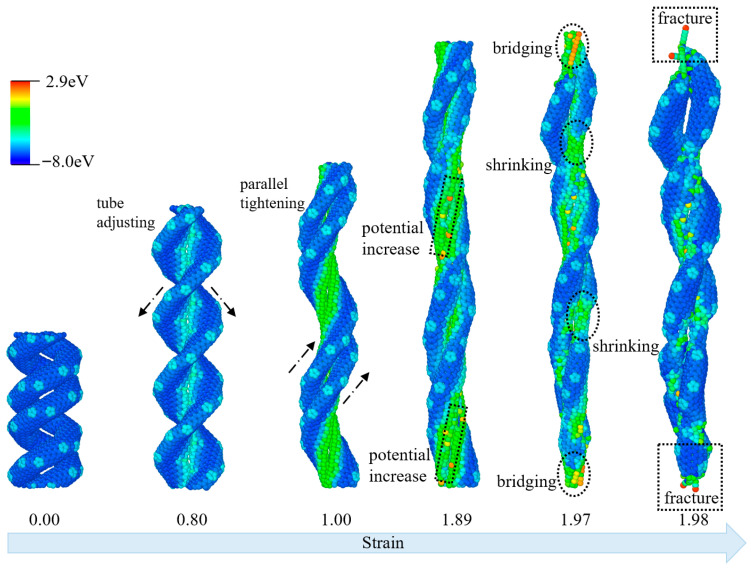
Structural evolutions of ECCNT1 during the tensile process. (The atoms are colored based on their potential energy.).

To better investigate the deformation behaviors of entwined CCNTs, the structural evolutions of ECCNT1 colored by per-atom potential energy during the tensile process are presented in Figure 6. The structural evolution of ECCNT1 under tensile load exhibits distinct characteristics at different stages. In the low strain range (up to 0.8), ECCNT1 primarily undergoes adjustments in tube structure. During this phase, the overall morphology of the ECCNT1 does not show significant stress concentration, and the atomic potential energy remains predominantly in a low-energy state, with no noticeable regions of increased potential energy. This stage corresponds to elastic deformation, where the structure experiences only micro-morphological fine-tuning. As the strain enters the medium range (0.80 to 1.89), the carbon nanotubes begin to exhibit parallel tightening, significantly enhancing the contact between the entwined tube chains. Concurrently, the atomic potential energy starts to increase, indicated by the expansion of green and yellow regions, and stress gradually concentrates at the contact sites of the entwined tubes. This marks the onset of plastic deformation, with interatomic interactions within the structure strengthening as the stress accumulates. When the strain further increases to the high range (1.89 to 1.97), ECCNT1 demonstrates characteristics of shrinking and bridging. Atoms in the entwined regions form local bridging structures to resist the tensile force, leading to the further expansion of high-potential energy regions and a more pronounced stress concentration effect. This stage represents a strengthening process driven by the unique entwining characteristics of the structure, which aims to delay the tensile failure. Finally, when the strain reaches 1.98, ECCNT1 enters the fracture stage. The ECCNT1 breaks at the sites of stress concentration, with the upper and lower sections connected only by chain-like structures, resulting in overall structural failure. Throughout the tensile process, atomic potential energy gradually increases with rising strain, and the regions of high potential energy closely coincide with the locations of structural deformation (tightening, shrinking, bridging) and final fracture. This observation reflects a direct positive correlation between stress concentration and changes in the atomic potential energy.

**Figure 7:**
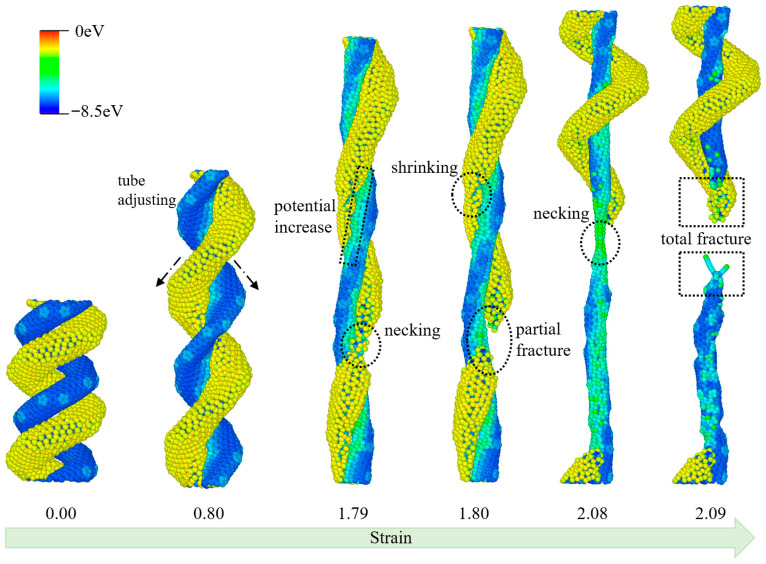
Structural evolutions of ECCNT2 during the tensile process. (The atoms are colored based on their potential energy.).

As illustrated in Figure 7, the tensile process of the ECCNT2 structure, composed of a hydrogenated carbon nanotube and a pristine carbon nanotube, exhibits distinct structural evolution and fracture characteristics as strain increases. At a strain of 0.00, the ECCNT2 structure maintains its initial entwined configuration with no obvious structural deformation, where the HCCNT and CCNT are closely intertwined. When the strain reaches 0.80, the nanotubes undergo adaptive adjustments in their relative positions within the entwined structure, accompanied by a slight increase in potential energy; however, no significant damage occurs to either the HCCNT or CCNT. At a strain of 1.79, necking behavior initiates in the HCCNT segment of the entwined structure, while the CCNT shows a tendency to shrink; simultaneously, the potential energy of the system rises notably, indicating the onset of structural stress concentration. A strain of 1.80 leads to the complete fracture of the HCCNT at the necking region, demonstrating that the hydrogenated modification renders the HCCNT more fragile in the entwined configuration compared to the pristine CCNT. As the tensile strain further increases to 2.08, pronounced necking occurs in the remaining CCNT component of the ECCNT2 structure, and partial fracture is observed at the necking site, signifying the progressive failure of the entwined system. Ultimately, at a strain of 2.09, the entire ECCNT2 structure experiences complete fracture at the CCNT’s necking region, marking the total collapse of the entwined carbon nanotube assembly under tensile loading.

**Figure 8:**
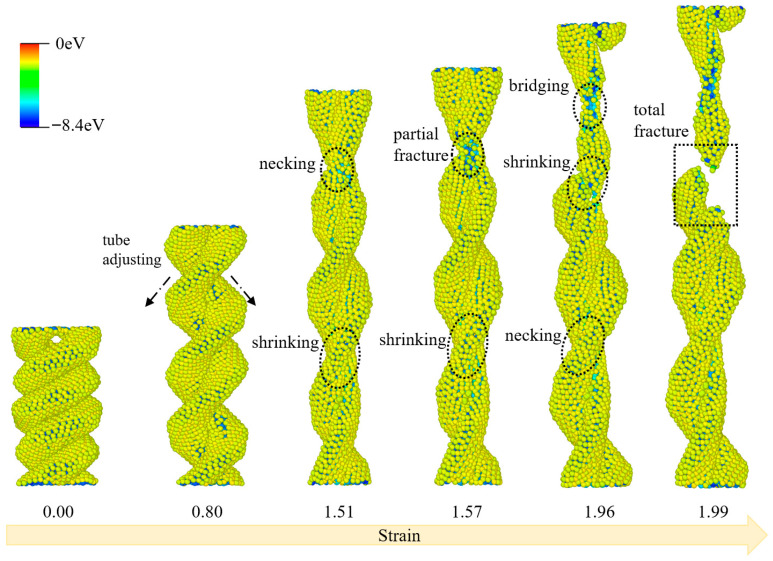
Structural evolutions of ECCNT3 during the tensile process. (The atoms are colored based on their potential energy.).

The tensile deformation process of ECCNT3 is characterized by a series of sequential structural changes, as illustrated in Figure 8. At an initial strain of 0.00, ECCNT3 maintains a regular and intact intertwined structure with uniformly distributed potential energy across the atomic framework. At a strain of 0.80, the nanotube undergoes tube adjustments accompanied by localized shrinking; this structural adaptation reflects the initial mechanical response of the intertwined architecture to tensile stress, with minor fluctuations in atomic potential energy emerging in the shrinking regions. As the strain increases to 1.51, pronounced necking occurs at the constricted sections of the CCNT, and the shrinking phenomenon becomes more prominent; the potential energy distribution exhibits noticeable heterogeneity at these deformed sites, indicating stress concentration within the structure. At a strain of 1.57, the nanotube experiences partial fracture at the necked regions, with persistent shrinking and secondary necking observed in adjacent areas; the potential energy of atoms at the fracture interface rises significantly, signifying the initiation of bond breakage in the intertwined carbon nanotube network. When the strain reaches 1.96, bridging forms between the fractured segments, and the nanotube undergoes further shrinking and necking; this bridging behavior temporarily mitigates the structural failure, demonstrating the toughening effect of the intertwined hydrogenated structure under tensile loading. Finally, at a strain of 1.99, total fracture occurs in ECCNT3, with the nanotube splitting into distinct segments at the most severely deformed region; the atomic potential energy at the fracture surface reaches the highest level throughout the entire tensile process, marking the complete failure of the intertwined carbon nanotube structure.

**Figure 9:**
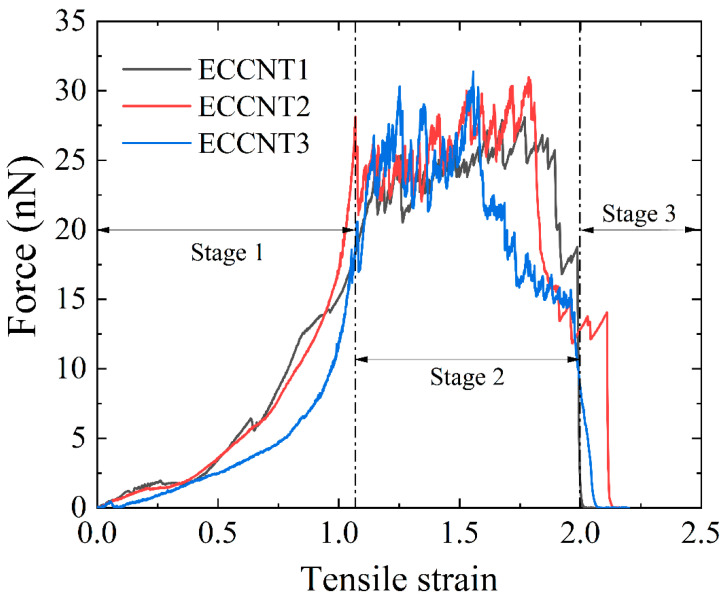
Force–strain curves of ECCNT1, ECCNT2, and ECCNT3 during the tensile process. Three distinct stages can be identified in the tensile processes of ECCNTs.

The force–strain curves of ECCNTs during the tensile process are presented in Figure 9. Three distinct stages can be identified in the tensile behavior of ECCNTs. All the ECCNTs show a rapid force rise in the increasing stage. It is evident that ECCNT1 and ECCNT2 exhibit a steeper slope during the increasing stage, indicating their higher tensile stiffness compared to the others. This can be attributed to the relatively higher stiffness of pristine CCNTs compared to HCCNTs, while ECCNT3 is composed of two HCCNTs with lower intrinsic stiffness. The oscillation stage features a slowdown in the force growth, followed by a peak and decline, reflecting plastic deformation and damage. In the oscillation stage, the peak tensile force of ECCNT3 reaches 31.4 nN, which is relatively higher than the 30.99 nN for ECCNT2 and 28.2 nN for ECCNT1. However, the tensile force of ECCNT2 drops rapidly at a strain of around 1.5, due to the fracture of one of the HCCNT structures, as mentioned in the previous section. At a strain of approximately 2.1, all the HCCNTs fracture, causing the tensile force to drop rapidly to zero. In the decrease stage, rapid fracture occurs with force plummeting to near zero. ECCNT2 demonstrates greater ductility compared to its counterparts, suggesting that the hybrid entwined nanospring contributes to the elongation of the ECCNT structures. Conversely, ECCNT1 exhibits less ductility, indicating that HCCNTs contribute more to the ductility of the entwined structures than CCNTs do.

The calculated tensile properties of different CCNTs and ECCNTs are presented in Table 2. It can be checked that CCNT1 obtains the highest spring constant, the elastic limit, and the maximum strain among all the single springs. It indicates that hydrogenation introduces hydrogen atoms that weaken the sp^2^ carbon–carbon bonds, reducing the stiffness of the CNT structure. HCCNT1 obtains the maximum force among the single springs, while HCCNT2 has the lowest one among them. It suggests that the partial hydrogenation is beneficial for the tensile resistance, while the full hydrogenation is harmful to the tensile properties of CCNTs. For the entwined CCNTs, entwining drastically enhances the mechanical performance of CCNTs: ECCNTs exhibit 1.5–2.0 times higher spring constants than that of the single CCNT/HCCNT, with ECCNT3 achieving the highest maximum force. Entwined structures show a slight decline in the elastic limit compared to the single ones, with a gradual reduction as the proportion of hydrogenated CNTs in the entwined system increases. In addition, ECCNTs achieve much higher maximum force than single ones, revealing that entwining drastically improves the tensile load capacity, and the combination of hydrogenated CNTs in the entwined system further increases the maximum force. ECCNT1 shows a moderate reduction in maximum strain relative to pure CCNT, while ECCNT2 maintains a strain close to CCNT and HCCNT1, balancing the stiffness and ductility. Overall, it can be concluded that entwining is a robust strategy to improve the tensile load capacity of coiled CNTs, and the incorporation of hydrogenated CNTs in entwined systems further optimizes load-bearing performance while slightly compromising elastic deformation capacity.

### 3.3. Energy Absorption

The energy absorption capacity of coiled carbon nanotubes during tensile deformation is quantitatively characterized by the total tensile energy absorption and specific energy absorption, which are derived from the tensile force–strain curves and atomic number of the models [36]. The total tensile energy absorption (*U*) is defined as the integral area under the force–strain curve from the initial state to the fracture strain, which reflects the total mechanical energy dissipated by the nanostructure during the entire tensile failure process. The specific energy absorption (*SEA*) is normalized by the total carbon atom number of the model, which eliminates the influence of atomic number differences on the energy absorption performance and enables a unified and fair comparison of energy absorption efficiency between individual CCNTs and entwined ECCNTs with different structural configurations. The corresponding calculation formulas are given as follows:

(7) $$U=L_{0}\int_{0}^{\varepsilon_{f}}F(\varepsilon )d\varepsilon$$

where *U* is the total tensile energy absorption, *F*(*ε*) is the fitting function of the tensile force–strain curve, and *L*_0_ is the initial axial length of the coiled nanotube, and *ε* is the fracture strain of the nanostructure at tensile failure.

(8) $$SEA=\frac{U}{N}$$

where SEA is the specific energy absorption, and *N* is the total number of carbon atoms in the CCNT/ECCNT model. The exclusion of non-load-bearing hydrogen atoms through this normalization approach is intended to enable a fair and consistent comparison among CCNTs and ECCNTs.

Based on the force–strain curves of individual CCNTs and entwined ECCNTs, the total tensile energy absorption and specific energy absorption of all six nanostructure models are calculated via numerical integration and normalization, and the corresponding results are summarized in Table 3.

For individual coiled structures, moderate hydrogenation (HCCNT1) improves both total energy absorption and specific energy absorption compared with the pristine CCNT. The enhanced distortion tolerance of the sp^2^–sp^3^ hybrid network enables more effective energy dissipation under tension, leading to a higher specific energy absorption of 0.048 eV per carbon atom. By contrast, full hydrogenation (HCCNT2) reduces both total and specific energy absorption, as the highly hydrogenated structure becomes brittle and fractures prematurely, limiting the overall energy dissipation capacity.

For entwined structures, entanglement significantly increases the total energy absorption compared with individual coils, owing to the enhanced inter-tube contact, frictional sliding, and hierarchical deformation mechanisms [2,4,35]. Among the entwined models, ECCNT2 exhibits the highest total energy absorption, benefiting from the balanced mechanical response of its hydrogenated–pristine hybrid configuration. In terms of specific energy absorption, ECCNT2 also maintains a high efficiency of 0.043 eV per carbon atom, outperforming both ECCNT1 and ECCNT3. ECCNT1 shows a slightly lower specific energy absorption, while ECCNT3 displays the lowest value due to excessive hydrogenation-induced brittleness.

Overall, these results demonstrate that moderate hydrogenation effectively enhances the energy absorption efficiency of individual CCNTs, while structural entanglement drastically improves the total energy absorption capacity. The hybrid entwined structure in ECCNT2 achieves the best overall performance by balancing high total energy absorption and favorable specific energy absorption efficiency, highlighting the synergistic effects of controlled hydrogenation and hierarchical entanglement.

## 4. Conclusions

This study systematically investigates the regulatory effects of hydrogenation and entanglement on the tensile properties and energy absorption capacity of CCNTs and their entwined derivatives using MD simulations, focusing on the interplay between hydrogenation and entanglement. The results confirm that hydrogenation exerts a dual regulatory role on individual CCNTs: HCCNT1 enhances tensile strength, total energy absorption, and specific energy absorption by improving the structural distortion tolerance of the sp^2^–sp^3^ hybrid network, and presents the strongest energy absorption efficiency among individual coils. In contrast, HCCNT2 induces severe brittleness, reducing ductility, total energy absorption, and specific energy absorption due to the complete sp^3^ hybridization of the carbon network. Additionally, entanglement significantly enhances the tensile load-bearing capacity and energy absorption performance of CCNTs: ECCNTs exhibit 1.5–2.0 times higher spring constants and total energy absorption than individual CCNTs/HCCNTs. This enhancement is attributed to synergistic inter-tube interactions, frictional sliding, and additional energy dissipation paths introduced by the hierarchical entwined structure, an effect that has not been fully clarified in previous studies on isolated CCNTs. Among the three ECCNT models, ECCNT2, composed of one hydrogenated CCNT and one pristine CCNT, achieves an optimal balance between tensile stiffness, ductility, total energy absorption, and energy absorption efficiency, outperforming ECCNT1 and ECCNT3 in overall energy absorption performance. In contrast, ECCNT3 exhibits the highest maximum tensile force but compromised ductility and the lowest specific energy absorption. These findings clarify the mechanistic roles of hydrogenation and entanglement in modulating the mechanical and energy absorption behaviors of CCNT-based nanostructures, providing valuable theoretical support and practical guidance for the rational design of high-performance hydrogenated ECCNT-based materials in flexible electronics, nanoscale actuators, and energy absorption fields.

## Figures and Tables

**Table 1:** Geometrical parameters of CCNTs and ECCNTs.

Type	Atom Number	Tube Radius (Å)	Effective Radius (Å)	Pitch Length (Å)
CCNT1	1440	4.0	10.8	31.5
HCCNT1	2160	5.0	11.0	31.5
HCCNT2	2880	5.0	11.0	31.5
ECCNT1	2880	4.0	10.8	15.8
ECCNT2	4320	4.5	11.0	15.8
ECCNT3	5760	5.0	11.0	15.8

**Table 2:** Summary of tensile properties of CCNTs/ECCNTs.

Type	Spring Constant (nN/nm)	Elastic Limit	Maximum Force (nN)	Maximum Strain
CCNT	1.76	1.18	16.36	2.16
HCCNT1	1.66	1.12	19.05	2.04
HCCNT2	1.62	0.92	15.99	1.84
ECCNT1	3.46	1.16	28.10	1.99
ECCNT2	3.17	1.06	30.98	2.11
ECCNT3	2.72	1.05	31.38	1.97

**Table 3:** Energy absorption properties of CCNTs and ECCNTs.

Type	Total Energy Absorption (eV)	Specific Energy Absorption (eV/Atom)
CCNT	64.54	0.045
HCCNT1	68.37	0.048
HCCNT2	58.90	0.041
ECCNT1	110.97	0.039
ECCNT2	123.89	0.043
ECCNT3	99.58	0.035

## Data Availability

The original contributions presented in this study are included in the article. Further inquiries can be directed to the corresponding author.
